# Nutrition and Periodontitis: A Cross-Sectional Study from a Practice-Based Research Network

**DOI:** 10.3390/nu16183102

**Published:** 2024-09-14

**Authors:** Stefanie Anna Peikert, Nils Benedikt Liedtke, Kirstin Vach, Eva Streletz, Steffen Rieger, Julia Palm, Felix Mittelhamm, Sebastian Kirchner, Peter Hakes, Laurence Gantert, Carmen Cansado De Noriega, Anne Brigitte Kruse, Petra Ratka-Krüger, Johan Peter Woelber

**Affiliations:** 1 Department of Dental Medicine and Oral Health, Medical University of Graz, Billrothgasse 4, 8010 Graz, Austria; 2 Department of Operative Dentistry and Periodontology, Faculty of Medicine, University of Freiburg, Hugstetter Straße 55, 79106 Freiburg, Germany; nils.liedtke@sund.ku.dk (N.B.L.); steffen.rieger@uniklinik-freiburg.de (S.R.); anne.kruse@uniklinik-freiburg.de (A.B.K.); petra.ratka-krueger@uniklinik-freiburg.de (P.R.-K.); 3 Section for Clinical Oral Microbiology, Department of Odontology, Faculty of Health and Medical Sciences, University of Copenhagen, Nørre Allé 20, 2200 Copenhagen N, Denmark; 4 Institute of Medical Biometry and Statistics, Faculty of Medicine and Medical Center, University of Freiburg, Stefan-Meier-Straße 26, 79104 Freiburg im Breisgau, Germany; kirstin.vach@uniklinik-freiburg.de; 5 Private Dental Practice Dr. Eva Streletz, 63150 Heusenstamm, Germany; dr.streletz@t-online.de; 6 Private Dental Practice Rieger Zahnmedizin, 72766 Reutlingen, Germany; 7 Private Dental Practice Dentapaz, 74072 Heilbronn, Germany; info@dentapaz.de; 8 Private Dental Practice Dres. Mittelhamm, 22399 Hamburg, Germany; praxis@zahnarzt-mittelhamm.de; 9 Private Dental Practice Die Dentistei, 72074 Tübingen, Germany; kontakt@die-dentistei.de; 10 Private Dental Practice Breisgaupraxis, 79206 Breisach am Rhein, Germany; info@breisgaupraxis.de; 11 Private Dental Practice Praxis auf der Allmend, 79822 Titisee-Neustadt, Germany; info@praxis-auf-der-allmend.de; 12 Private Dental Practice CADENO Dental Concepts, 6850 Dornbirn, Austria; c.cansado@cadeno.at; 13 Clinic of Operative Dentistry, Periodontology, and Pediatric Dentistry, Medical Faculty Carl Gustav Carus, Technische Universität Dresden, 01069 Dresden, Germany; johan.woelber@ukdd.de

**Keywords:** dietary behavior, periodontitis, practice-based research network, PBRN

## Abstract

Background: Despite clinical interventional studies on the influence of diet on periodontal inflammatory parameters, there has been no practice-based cross-sectional study from a German population to date that has conducted both a comprehensive dental and periodontal examination and a thorough validated assessment of dietary behavior. Therefore, the aim of this pilot study was to evaluate, in a proof of concept, whether there is a correlation between the overall periodontal inflammatory surface area (PISA), periodontal clinical parameters (pocket probing depths (PPD), clinical attachment loss (CAL), bleeding on probing (BOP), furcation involvement (FI), tooth mobility (TM)), and the dietary behavior of patients with periodontal disease when utilizing a practice-based research network. The primary outcome was the correlation between the periodontal inflammatory surface (PISA) and the dietary assessment data. Materials and Methods: The practice-based research network, consisting of eight Master’s graduates, recruited patients who met the inclusion and exclusion criteria and performed a periodontal examination together with the assessment of dietary behavior using a digital version of the validated retrospective dietary recall (DEGS/RKI). Statistical analyses included linear regression models adjusted for age and smoking and unpaired *t*-tests, conducted using STATA 17.0 with a significance level of 5%. In addition, the data obtained were classified according to the currently recommended amounts of daily intake. Results: A total of 1283 teeth were analyzed, with 60.25% (773 teeth) requiring treatment. The average PISA was 753.16 mm^2^ (SD ± 535.75 mm). Based on dietary guidelines, the studied population consumed excessive amounts of extrinsic sugars and fats, while their fiber and legume intake was insufficient. The intake of certain nutrients, including water-soluble fibers, specific fatty acids, vitamins (D, B1, B2, B6, and B12), iron, and zinc, was associated with reduced PISA, PPD, CAL, and BOP. Conclusion: Within the limits of the current study, including its cross-sectional design and cohort size, the outcomes demonstrated the influence of nutrition on periodontal health.

## 1. Background

In the past, gingival inflammation and the subsequent development of periodontitis were predominantly ascribed to oral biofilm [1]. Consequently, therapeutic strategies have largely focused on the elimination or regulation of this oral biofilm through chemomechanical methods [2]. However, recent research has increasingly highlighted significant variations in oral biofilm composition among individuals, including the presence of various oral pathogens in healthy individuals without apparent disease symptoms [1,2]. Moreover, it has been recently demonstrated that the host organism must initially provide a suitable colonization base for certain pathogenic microorganisms through inflammatory reactions to eventually lead to disease manifestation [3]. Systemic diseases like diabetes, cardiovascular diseases, and metabolic syndrome are also influencing periodontal health by sharing etiopathogenetic pathways [4]. Besides genetic and systemic factors, nutrition appears to have a considerable impact on both systemic and periodontal inflammatory processes [5,6,7,8]. These studies have revealed that a diet high in processed carbohydrates (such as sugar and refined flour), saturated fats, and trans fats and low in micronutrients, fiber, and omega-3 fatty acids has pro-inflammatory effects. These dietary components, characteristic of the prevalent Western diet in industrialized nations, have been linked to systemic inflammatory responses in numerous studies [9,10,11,12]. Furthermore, Kotsakis et al. (2017) demonstrated, using data from the US National Health and Nutrition Examination Surveys (NHANES), that a pro-inflammatory diet was significantly correlated with tooth loss [13]; however, this study did not conduct a detailed periodontal examination, thus, the impact of specific dietary patterns on various clinical periodontal parameters remains unclear. Therefore, the hypothesis of the present study is that the consumption of pro-inflammatory foods is associated with greater periodontal destruction compared to micronutrient-rich and fiber-rich foods.

Traditionally, scientific and clinical research is conducted within academic institutions. However, it is estimated that only 1% of patients in the United States receive treatment in such institutions [14]. This means that the participants who are willing to take part in the research vary between the academic and private sectors. Apart from the aforementioned clinical interventional studies on the influence of nutrition on periodontal inflammatory parameters, no cross-sectional study from a German population has been conducted with both a comprehensive dental and periodontal examination (including dental assessment, pocket probing depths, clinical attachment levels, bleeding on probing, tooth mobility, and furcation involvement) and a thorough validated assessment of dietary behavior [15]. Accordingly, this study aimed to establish a practice-based research network for the collection of periodontal data and to assess the nutritional behavior of patients with periodontal disease.

## 2. Materials and Methods

### 2.1. Data Collection

This non-interventional, retrospective, observational pilot study, approved by the Ethics Committee of the University of Freiburg (ETK-FR No. 30/19), was registered in the German Clinical Trials Registry (DRKS00034554) and funded by the German Research Foundation (PE 3124/1-1) and domestic funds. A practice-based research network was established, involving eight students or graduates from the Master’s program in Periodontology and Implant Therapy at the University of Freiburg and the study center [16]. Consequently, they were trained using a standard protocol based on approved guidelines to ensure a consistent level of treatment and education. Practices received detailed information about this non-interventional observational study and provided informed consent. They all routinely used a digital periodontal examination program (ParoStatus Version X, Parostatus.de GmbH, Berlin, Germany) for documentation. Practitioner recruitment took place between February and June 2021. In September 2021, an initial meeting provided a detailed study overview and addressed questions. Following the start of the practice-based research network, patients with periodontitis were recruited. Those requiring systematic periodontitis therapy, as indicated by the Community Periodontal Index of Treatment (CPITN), and meeting the selection criteria, were eligible for inclusion [17].

The selection criteria were defined as the following:

- Patients aged 18 years or older;
- No antibiotic use in the 6 months prior to or during the study;
- Scheduled for periodontal therapy;
- No systematic periodontal treatment in the previous 24 months.

Patient recruitment occurred between October 2021 and February 2023. Systematic periodontal assessment included a full-mouth periodontal status, involving pocket probing depths (PPD), clinical attachment loss (CAL), bleeding on probing (BOP), tooth mobility (TM), and furcation involvement (FI).

The validated DEGS nutrition questionnaire, a food frequency questionnaire used in the DEGS 1 study from 2008 to 2011, was employed to assess dietary behavior [18]. The digital DEGS nutrition questionnaire was implemented in collaboration with ParoStatus.de GmbH. Patients completed the questionnaire in the waiting room, on a practice device, or at home via a web link or QR code. Data transmission required a computer with ParoStatus software (Parostatus Version X, Parostatus.de GmbH, Berlin, Germany) and a mobile device with the according app. Data were not stored online during questionnaire completion, ensuring privacy by restricting storage to the patients and the practice.

Pseudonymization and data conversion were performed by the Parostatus software, directly associating nutritional questionnaires with periodontal findings [19].

### 2.2. Nutritional Analysis

Nutritional analysis of the dietary questionnaires was conducted using Microsoft Excel 2016 (Microsoft, Redmond, WA, USA) and EbisPro 2016 (University of Hohenheim-Stuttgart, Germany). Average daily intake for all 53 food items over the past four weeks was calculated based on reported quantities and frequencies. Nutritional information was primarily sourced from the EbisPro software, with sugar content calculated as the difference between total carbohydrates and starch. These values were verified and supplemented using nutritional tables by Heseker and Heseker (2021) and the Swiss Food Composition Database (Federal Food Safety and Veterinary Office FSVO, 2023) [20,21]. Attention was given to nutrients affecting periodontitis, including carbohydrates, intrinsic sugars, dietary fibers, and omega-3 fatty acids, based on a previous randomized clinical trial. Foods were categorized into different groups following Haftenberger et al. (2010) [18]. Subsequently, the Excel workbooks were forwarded to the Institute of Medical Biometry and Statistics (IMBI) for further statistical analysis.

### 2.3. Statistical Procedures

The primary outcome was the correlation between the total periodontal inflammation surface area (PISA) and the questionnaire data. The correlation between different food groups and periodontal clinical parameters (pocket probing depths (PPD), clinical attachment loss (CAL), bleeding on probing (BOP), furcation involvement (FI), and tooth mobility (TM)) was considered a secondary outcome criterion. For the descriptive analysis, median, mean, standard deviation, minimum, and maximum values were calculated. Linear regression analysis was performed between the clinical parameters (PPD, CAL, BOP, FI, TM, and tooth loss without wisdom teeth) and diet by using models adjusted for age and smoking status [22,23,24,25]. The descriptive evaluation of nutritional data was compared with reference values from the German Nutrition Society and the World Health Organization to identify any nutrient over- or under-supply in the study population [26,27], with positive and negative deviations calculated accordingly The classification of foods as pro-inflammatory or anti-inflammatory in Table 5 was based on the guidelines provided in the paper of Shivappa et al. (2014) [28]. The classification into moderate and severe periodontitis was based on Eke et al. (2012) [29]. Subsequently, group differences between moderate and severe periodontitis were determined using an unpaired *t*-test. Linear regression models with adjustments for age and smoking were used to evaluate the influence of several nutrients on the clinical parameters. All data were analyzed using STATA 17.0 (StataCorp LLC, College Station, TX, USA). The level of statistical significance was set to 5%. Due to the explorative design of the study, no sample size calculation and no adjustment for multiple testing was carried out.

## 3. Results

Eight Master’s graduates from Germany and Austria were recruited for data collection, with collaboration also involving the Periodontology Department of the Freiburg University Medical Center. These graduates conducted data collection on a total of 60 patients who met the specified inclusion and exclusion criteria. In the final analysis, 50 data sets were included. The exclusion criteria for patients were primarily due to the use of antibiotics within the six months prior to data collection (five dropouts), previous periodontal treatments within the last two years (four dropouts), and invalid periodontal findings such as consistent probing depths of 0 mm (one dropout).

Among the examined patients, 13 exhibited moderate periodontitis (MP) while 37 were diagnosed with severe periodontitis (SP). The descriptive data of the sample are shown in Table 1.

A total of 1283 teeth were analyzed. Of these, 59.97% required treatment (probing depth ≥ 4 mm with bleeding). The average inflammatory surface area, measured as the Periodontal Inflamed Surface Area (PISA), was 753.16 mm^2^ (SD ± 535.75 mm^2^). The mean bleeding on probing (BOP) index was 32.85%. The average probing depth was 3.05 mm, accompanied by recessions of −0.52 mm, resulting in a clinical attachment loss (CAL) of 3.57 mm. All values of patient-specific clinical parameters are listed in Table 2.

Regarding the nutritional data, a total of 40 nutrients and 27 food categories were analyzed. 

Subsequently, the correlations between the periodontal inflammatory surface area (PISA), periodontal clinical parameters (PPD, CAL, BOP, FI, and TM), and various food groups were examined. Daily quantity intake was included in the regression analyses (Table 3).

It was observed that the intake of water-soluble fibers, medium-chain fatty acids, carotene, Vitamin D, Vitamins B1 and B2, iron, and zinc as well as butter and foods containing extrinsic sugars were associated with a reduced PISA area (Table 3).

Similar trends were observed in the results of the regression analyses for probing depths (PPD). Here, the intake of water-soluble fibers, medium-chain fatty acids, carotene, Vitamins B1, B2, B6, and B12, iron, and zinc led to lower PPD values.

Regarding clinical attachment loss (CAL), patients who consumed water-soluble fibers, Vitamins B1, B2, and B6, pantothenic acid, iron, and zinc exhibited reduced CAL.

For bleeding on probing (BOP), negative correlations were found with short-chain and medium-chain fatty acids and Vitamins B1 and B6.

The clinical parameter of teeth requiring treatment also showed a relation between fiber, Vitamin A, Vitamin E, folic acid, Vitamin C, and vegetables.

Furcation involvement (FI) and tooth mobility (TM) showed no statistically significant associations in the regression analysis, although tendencies can be observed like those shown in Table 3.

In addition to the observed correlations between clinical parameters and nutrition, the mean values were presented for the entire population as well as separately for the subgroups of patients with moderate and severe periodontitis. The daily intake of the nutrient groups in patients with moderate and severe periodontitis was compared using a *t*-test (Table 4).

The average daily energy intake of the studied population was 1819 kcal (SD ± 1574). For macronutrients, the average daily intake was 78 g of protein, 65 g of fat, and 212 g of carbohydrates, of which approximately 111 g were sugars (including 36 g of extrinsic (free) sugars and 75 g of intrinsic sugars). The average daily fat intake was 65 g.

The daily fiber intake was 21 g. The total water intake, including beverages, was 4342.28 g, with actual water consumption being 2278.21 g.

Patients with moderate periodontitis had a higher average daily energy intake of 2283.92 kcal, 37.96% higher than the 1655.45 kcal in the severe periodontitis group. Differences in dietary habits between the two groups included lower consumption of butter, less bread, and a preference for whole-grain products among patients with moderate periodontitis. Additionally, these patients consumed fewer sweet spreads and more fruit. Their fiber intake was also higher (28.75 g, SD ± 20.64) compared to those with severe periodontitis (18.24 g, SD ± 13.07).

The moderate periodontitis group also had a 19.06% higher fat intake and consumed significantly more intrinsic sugars (68.54%) and fruit (134.92%). Notably, the intake of Vitamin C (*p* = 0.018), Vitamin B6 (*p* = 0.039), Biotin (*p* = 0.040), potassium (*p* = 0.047), tea (*p* = 0.035), cereals (*p* = 0.001), and fruit (*p* = 0.0032) was significantly higher in the moderate periodontitis group compared to the severe periodontitis group.

The comparison of all patients—including the moderate and severe periodontitis groups—with the recommended daily intake from the German Nutrition Society and the WHO is shown in Table 5. 

In the total population, it can be seen that more saturated fatty acids were consumed than recommended, whereas the intake of polyunsaturated fatty acids was insufficient. According to the dietary guidelines, the recommended intake of extrinsic sugars was exceeded by 59.06%. Except for Vitamins D and E, and folic acid, the vitamin requirements were met with the current diet. While enough fruit was consumed, the consumption of fish and vegetables did not meet the recommended reference values While the MP group had a higher daily intake of energy compared to the recommendations, a daily intake in fat below the recommendations was observed. The group of SP, on the other hand, showed opposite values. Daily fiber intake was in both groups below the recommendations. However, the MP groups’ values deviated only slightly from the recommendation. It can be shown that the group of SP has a deficiency in the intake of Vitamin E and Iodide while the group of MP met the recommendations.

An increased intake in the MP group and decreased intake in the SP group compared to the recommendations was shown for folic acid, potassium, fish, fruit, and the food category rice/noodles/potatoes.

While the daily recommendations for Vitamin B6, Biotin, Vitamin C, and Sodium were met by the SP group, the MP group showed a higher intake compared to the SP group. In addition, although saturated fatty acids intake was above the recommended values, the MP group showed lower overconsumption.

## 4. Discussion

The goal of this study was to explore the feasibility of gathering nutrition data alongside periodontal data within a practice-based research network. The data collection was successful, and the participating practices showed strong motivation to contribute to the study. The nutritional data were seamlessly integrated with the periodontal assessment data, with the extraction of periodontal findings from the Parostatus program for statistical analysis already well-established in previous studies [30]. Consequently, this pilot study lays the groundwork for subsequent studies with larger patient cohorts.

### 4.1. Clinical Periodontal Data

According to the hypothesis of this study, significant correlations between the total inflammation area (PISA) and the dietary behavior—as well as between the clinical findings of pocket probing depth (PPD), clinical attachment loss (CAL), bleeding on probing (BOP), furcation involvement (FI), and tooth mobility (TM)—was shown. A correlation between the number of teeth lost and dietary behavior could not be confirmed. In view of the sample of 50 patients, further follow-up studies are required to confirm the results. Like in previous studies, it was shown that it is possible to collect clinical periodontal data within a practice-based research network.

In the present sample, there were significantly more patients with severe periodontitis (SP) in relation to moderate periodontitis (MP) compared to the Fifth German Oral Health Study (DMS V) [31]. The present patient population therefore does not correspond to the average population of a regular dental practice in terms of the severity of periodontitis. This discrepancy may be due to the specialization of the participating practices in the field of periodontology.

### 4.2. Nutrition Data

The population’s daily energy intake of 1819 kcal was 19% less than the recommended average intake of 2250 kcal [26].

Daily energy intake was negatively associated with the clinical outcomes of PPD, CAL, and BOP. The influence and significance of the daily energy intake on periodontal diseases can be discussed in various ways. According to van Woudenbergh et al. (2013), the energy parameter is merely a summary of all parameters with regard to inflammatory conditions [32]. However, if excessive energy intake results in obesity, this can become a significant risk factor for periodontitis [33,34,35].

The recommended daily fiber intake of 30 g could not be achieved with a deficit of 30% and an average value of 21 g. Compared to the German National Nutrition Survey II (NVS II), the daily intake of dietary fiber was stated at 27.3 g for men and 26.1 g for women [36]. The significant differences in fiber intake between the moderate and severe periodontitis groups (*p* = 0.038) in this study confirmed Merchant et al.’s (2006) findings that increased fiber intake is associated with a reduced risk of periodontitis [37].

Highly processed carbohydrates such as free sugars are often associated in the literature as pro-inflammatory and with a negative impact on periodontal health [38]. The WHO puts the maximum recommended level of free sugars at 10% of daily energy intake but recommends less than 5% [27]. In the present study, 144 kcal of free sugars were consumed daily. This corresponds to an overconsumption of 58% according to the daily amount recommended by the WHO. It should also be highlighted that the group with moderate periodontitis consumed almost twice as much fruit compared to the severe periodontitis group. This could be the reason why the moderate periodontitis group consumed significantly more intrinsic sugars (*p* = 0.016).

Furthermore, it was demonstrated that the moderate periodontitis group consumed significantly more Vitamin C (*p* = 0.018). A significant association between periodontal disease and lower dietary vitamin C intake was also shown in other studies [39,40].

Vitamin B6 intake also showed significant differences (*p* = 0.039) between the two groups, while its consumption was higher in the moderate periodontitis group. Other studies have also shown that there is a correlation between the risk of periodontitis and a lower intake of Vitamin B [41,42]. A significantly higher intake of Biotin (*p* = 0.04) and Potassium (*p* = 0.047) in the moderate periodontitis group was also observed. The average intakes of Biotin and Potassium in the moderate periodontitis group were higher than the recommended daily intake, while the daily intakes in the severe periodontitis group were within the recommended values [26].

Overall, by distinguishing nutrients and food groups, this provides an opportunity to address different interests and approaches in nutrition research. Nutrients can provide information about the causality of nutritional behavior on an immunological level. Food groups, on the other hand, have the advantage of visualizing the patient’s actual dietary habits. This summary can be a good way of explaining diets in a simplified and logical way, especially in nutritional counseling, to initiate practical changes that can be implemented by the patients. The findings confirmed that an anti-inflammatory diet is associated with the periodontal health of patients. In the context of periodontal treatment and maintenance therapy, nutrition should be included in the treatment concept as a modifying factor. The questionnaire used here can facilitate patient communication and make dietary changes visible to the patient over time.

### 4.3. Limitations 

A fundamental limitation of this study can be seen in the cross-sectional design, which does not allow any causality between dietary behavior and the extent of periodontitis as the study does not provide any information on the causes of the different diets. Furthermore, as we only had access to clinical data and lacked radiographic data or information on the need for complex rehabilitation, it was not possible to accurately assess the complexity of the disease according to the current staging classification [43]. Therefore, we used the classification of Eke et al. (2012) [29], which is based solely on clinical data.

Besides that, there have been no corrections regarding the multiple testing of parameters. Given the large number of tests conducted, significant results could be expected; therefore, the results should be critically assessed for statistical significance. However, the number of significant results exceeds the expected number. A closer look at the nutritional data revealed that no trans fatty acids were considered in calculating the daily nutritional values and food groups. Beyond that, the quality of carbohydrates (processed/whole grain) could only be recorded for bread, despite a wide selection of whole-grain alternatives for rice and noodles available on the market. When it comes to dairy products, some alternatives to animal products were mentioned but do not represent the available products. The RKI questionnaire is a validated and frequently used FFQ nutrition questionnaire [18]. In the present study, it was quite easy to implement the questionnaire compared to other methods of dietary assessment like a 24-hour survey method. This method was used in the German National Nutrition Survey II (NVS II), recording the dietary behavior of the last 24 h by trained interviewers at two points in time using open questions [44]. Using an FFQ nutrition questionnaire, which covers the last four weeks of dietary habits, provides the possibility to also record less-often consumed foods; however, it requires good memory performance for recalling the last four weeks. Beyond that, it is time- and resource-efficient because no trained interviewers are needed, and patients are able to fill out the questionnaire at different locations.

The total inflammation surface (PISA), recorded in patients with severely reduced residual dentition should also be mentioned. The PISA only provides information about the inflamed epithelial surface. Patients with little remaining dentition have significantly less epithelium that can become inflamed due to the smaller possible inflamed epithelial surface. The PISA is well-suited for overall inflammation in relation to the systemic effect as well as for monitoring progression; however, it is only of limited use for assessing the severity of periodontitis.

### 4.4. Practical Implications

This study highlights the importance of integrating nutritional counseling into periodontitis treatment as the data support its positive impact on patient outcomes. Additionally, these findings advance practice-based research by enabling the generation of large data sets without disrupting clinical workflows, providing a more representative cohort, and supporting personalized medicine through the integration of periodontal and general medical data.

### 4.5. Perspective

It has been shown that high-quality research with large cohorts of patients can also be conducted outside academic institutions. This study also demonstrated a significant correlation between the intake of specific foods and a notable reduction in periodontal destruction.

In addition to the documentation of nutritional behavior and periodontal findings, serological parameters for further assessment would be advantageous alongside a detailed medical history.

The strength of practice-based research networks lies not only in the size of their cohorts but also in the size of the data set of individual patients.

## 5. Conclusions

Within the limits of the current study, including its cross-sectional design and cohort size, it was shown that it is feasible and scalable to collect periodontal data in combination with other relevant healthcare data within a practice-based research network. Furthermore, it was demonstrated that anti-inflammatory nutrition was significantly associated with better periodontal health. Due to the small sample size, further follow-up studies are required to confirm the results.

## Figures and Tables

**Table 1:** Descriptive analysis and distribution of classification of the total population.

Gender	N (%)	Age in Years	Smoking (%)	Diabetes (%)	Moderate Periodontitis (%)	Severe Periodontitis (%)
female	28 (56)	54.59	8 (16)	2 (4)	8 (16)	20 (40)
male	21 (42)	63.42	2 (4)	2 (4)	4 (8)	17 (34)
other	1 (2)	40	0 (0)	0 (0)	1(2)	0 (0)
total	50 (100)	58	11 (20)	4 (8)	13 (26)	37 (74)

**Table 2:** Mean values (±SD) of patient-specific clinical parameters.

Parameter	Mean (±SD)
PPD [mm]	3.05 (±0.64)
CAL [mm]	3.57 (±1.26)
BOP [%]	32.85 (±21.61)
Teeth requiring treatment [%]	59.97 (±28.52)
PISA [mm^2^]	753.16 (±535.75)

**Table 3:** Results of regression analysis investigating the influence of the different food groups on different clinical parameters. Regression coefficient (with *p*-value in parentheses). Columns marked with * were multiplied by 100 for better clarity. The significant values are printed in bold.

Food Group			Regression				
	PISA	PPD *	CAL *	BOP *	FI *	TM *	Teeth Requiring Treatment *
Energy [kcal]	**−0.104 (0.016)**	**−0.017 (0.002)**	**−0.027 (0.014)**	**−0.004 (0.023)**	−0.006 (0.386)	−0.002 (0.236)	**−0.005 (0.044)**
Water [g]	−0.022 (0.506)	−0.008 (0.073)	−0.013 (0.114)	0.018 (0.615)	0.001 (0.686)	−0.001 (0.5)	−0.002 (0.17)
Protein [g]	**−2.639 (0.025)**	**−0.45 (0.003)**	**−0.597 (0.047)**	**−0.105 (0.031)**	−0.18 (0.258)	−0.046 (0.247)	**−0.131 (0.037)**
Fat [g]	**−2.686 (0.022)**	**−0.378 (0.013)**	−0.374 (0.218)	**−0.109 (0.024)**	−0.216 (0.141)	−0.047 (0.23)	−0.111 (0.079)
Carbohydrates [g]	**−0.843 (0.018)**	**−0.158** **(<0.001)**	**−0.281 (0.001)**	**−0.032 (0.03)**	−0.016 (0.772)	−0.013 (0.29)	**−0.042 (0.03)**
Sugar total [g]	**−1.642 (0.038)**	**−0.281 (0.005)**	**−0.519 (0.009)**	−0.052 (0.11)	−0.032 (0.704)	−0.035 (0.177)	−0.07 (0.098)
Intrinsic sugar [g]	−2.213 (0.061)	**−0.453 (0.002)**	**−1.033 (<0.001)**	−0.072 (0.141)	0.057 (0.671)	−0.039 (0.315)	−0.119 (0.06)
Extrinsic sugar [g]	−2.356 (0.122)	−0.282 (0.156)	−0.197 (0.615)	−0.073 (0.247)	−0.176 (0.243)	−0.064 (0.2)	−0.061 (0.456)
Fiber [g]	**−9.326 (0.03)**	**−1.971** **(<0.001)**	**−3.733** **(<0.001)**	**−0.369 (0.037)**	0.585 (0.285)	−0.104 (0.472)	**−0.542 (0.018)**
Starch [g]	**−1.227 (0.031)**	**−0.243 (0.001)**	**−0.422 (0.003)**	**−0.052 (0.026)**	−0.003 (0.981)	−0.013 (0.499)	**−0.066 (0.03)**
Water-soluble fiber [g]	**−30.212 (0.024)**	**−6.469** **(<0.001)**	**−11.806** **(<0.001)**	**−1.232 (0.025)**	2.386 (0.204)	−0.314 (0.484)	**−1.841 (0.009)**
Water-insoluble fiber [g]	**−13.985 (0.029)**	**−3.008** **(<0.001)**	**−5.552** **(<0.001)**	**−0.566 (0.031)**	0.872 (0.285)	−0.143 (0.505)	**−0.857 (0.011)**
Saturated fatty acids [g]	**−5.356 (0.028)**	**−0.635 (0.046)**	−0.33 (0.603)	**−0.214 (0.032)**	−0.384 (0.145)	−0.094 (0.249)	−0.205 (0.12)
Polyunsaturated fatty acids [g]	**−14.259 (0.038)**	**−2.744 (0.002)**	**−4.706 (0.006)**	**−0.577 (0.041)**	−1.67 (0.258)	−0.216 (0.347)	−0.71 (0.054)
Short-chain fatty acids [g]	**−72.754 (0.047)**	−7.709 (0.108)	2.694 (0.776)	**−2.988 (0.047)**	−4.72 (0.184)	−1.052 (0.389)	−3.555 (0.07)
Medium-chain fatty acids [g]	**−95.369 (0.018)**	**−12.103 (0.021)**	−5.998 (0.569)	**−3.964 (0.017)**	−5.995 (0.162)	−1.257 (0.354)	**−4.693 (0.03)**
Long-chain fatty acids [g]	**−3.077 (0.022)**	**−0.439 (0.011)**	−0.463 (0.181)	**−0.124 (0.024)**	−0.254 (0.139)	−0.054 (0.225)	−0.126 (0.081)
Alcohol [g]	**−8.272 (0.04)**	−0.813 (0.124)	**−2.754 (0.006)**	−0.302 (0.069)	−0.081 (0.9)	−0.177 (0.187)	−0.106 (0.629)
Vitamin A [µg]	**−0.14** **(0.01)**	**−0.023 (0.001)**	−0.023 (0.096)	**−0.006 (0.012)**	0.007 (0.274)	−0.001 (0.561)	**−0.008 (0.005)**
Carotene [mg]	**−24.059 (0.024)**	**−4.153 (0.002)**	−5.164 (0.059)	**−0.956 (0.03)**	2.199 (0.08)	−0.085 (0.813)	**−1.478 (0.009)**
Vitamin D [µg]	**−26.069 (0.041)**	−3.191 (0.054)	−6.241 (0.054)	**−1.076 (0.039)**	−2.41 (0.223)	−0.547 (0.196)	−0.906 (0.188)
Vitamin E (equivalent) [mg]	**−15.599 (0.02)**	**−2.875 (0.001)**	**−4.533 (0.007)**	**−0.645 (0.02)**	−0.27 (0.804)	−0.184 (0.416)	**−0.847 (0.018)**
Vitamin B_1_ [mg]	**−193.057 (0.036)**	**−40.987 (<0.001)**	**−65.828 (0.004)**	**−7.629 (0.044)**	4.054 (0.763)	−3.132 (0.309)	**−11.147 (0.023)**
Vitamin B_2_ [mg]	**−117.48 (0.044)**	**−18.152 (0.016)**	−27.378 (0.066)	−3.469 (0.152)	−6.858 (0.266)	−2.766 (0.153)	−5.501 (0.079)
Niacin [mg]	−9.834 (0.068)	**−2.122 (0.002)**	**−4.279 (0.001)**	−0.282 (0.206)	−0.326 (0.733)	−0.179 (0.316)	−0.53 (0.065)
Pantothenic acid [mg]	**−38.94 (0.024)**	**−7.571** **(<0.001)**	**−13.591 (0.001)**	−1.375 (0.054)	−0.599 (0.792)	−0.699 (0.225)	**−2.152 (0.019)**
Vitamin B_6_ [mg]	**−109.342 (0.032)**	**−23.468 (<0.001)**	**−45.328** **(<0.001)**	−4.008 (0.057)	4.463 (0.569)	−1.53 (0.371)	**−6.218 (0.022)**
Biotin [µg]	**−4.444 (0.026)**	**−0.817 (0.001)**	**−1.449 (0.003)**	−0.153 (0.064)	−0.048 (0.832)	−0.094 (0.158)	**−0.244 (0.021)**
Folic acid [µg]	**−0.857 (0.015)**	**−0.153 (0.001)**	**−0.228 (0.01)**	**−0.033 (0.024)**	<0.001 (0.995)	−0.012 (0.296)	**−0.049 (0.009)**
Vitamin B_12_ [µg]	**−32.896 (0.048)**	**−5.819 (0.006)**	−7.268 (0.086)	−1.319 (0.054)	−3.943 (0.086)	−0.644 (0.243)	**−1.74** **(0.05)**
Vitamin C [mg]	−0.902 (0.061)	**−0.202 (0.001)**	**−0.387 (0.001)**	−0.033 (0.096)	0.089 (0.096)	−0.008 (0.61)	**−0.06 (0.018)**
Sodium [mg]	**−0.058 (0.033)**	**−0.011 (0.001)**	**−0.018 (0.01)**	**−0.002 (0.028)**	−0.005 (0.414)	0.001 (0.387)	**−0.003 (0.034)**
Potassium [mg]	**−0.062 (0.025)**	**−0.012** **(<0.001)**	**−0.024** **(<0.001)**	−0.002 (0.066)	<0.001 (0.909)	0.001 (0.303)	**−0.003 (0.027)**
Calcium [mg]	−0.161 (0.056)	−0.019 (0.086)	−0.014 (0.517)	−0.005 (0.163)	−0.01 (0.219)	−0.003 (0.283)	−0.007 (0.103)
Magnesium [mg]	**−0.605 (0.039)**	**−0.116 (0.002)**	**−0.225 (0.002)**	−0.016 (0.191)	−0.004 (0.92)	−0.012 (0.237)	**−0.031 (0.049)**
Phosphorus [mg]	**−0.161 (0.02)**	**−0.025 (0.005)**	−0.033 (0.064)	**−0.006 (0.035)**	−0.01 (0.209)	−0.003 (0.22)	**−0.008 (0.041)**
Iron [mg]	**−16.737 (0.047)**	**−3.101 (0.004)**	**−6.077 (0.004)**	−0.608 (0.08)	−0.099 (0.939)	−0.313 (0.264)	−0.686 (0.131)
Zinc [mg]	**−18.507 (0.041)**	**−3.412 (0.003)**	**−5.469 (0.017)**	**−0.741 (0.047)**	−1.213 (0.379)	−0.331 (0.274)	−0.909 (0.061)
Iodine [µg]	−1.41 (0.057)	**−0.24 (0.011)**	**−0.401 (0.032)**	−0.043 (0.158)	−0.097 (0.289)	−0.029 (0.235)	−0.07 (0.076)
Cholesterol [mg]	−0.604 (0.052)	**−0.099 (0.013)**	−0.139 (0.078)	**−0.026 (0.045)**	−0.046 (0.262)	−0.014 (0.17)	−0.028 (0.92)
Tea [g]	0.035 (0.692)	−0.008 (0.462)	−0.006 (0.777)	0.002 (0.623)	−0.006 (0.5)	< 0.001 (0.973)	−0.002 (0.622)
Coffee [g]	−0.034 (0.83)	0.003 (0.878)	0.001 (0.983)	0.008 (0.224)	−0.005 (0.744)	−0.004 (0.495)	**0.979** **(<0.001)**
Non-alcoholic beverages [g]	−0.221 (0.304)	−0.049 (0.073)	−0.031 (0.566)	−0.007 (0.399)	−0.019 (0.373)	−0.006 (0.428)	−0.019 (0.1)
Water as a beverage [g]	0.028 (0.545)	0.001 (0.874)	0.001 (0.96)	0.003 (0.1)	0.006 (0.215)	< 0.001 (0.95)	**0.949** **(<0.001)**
Alcoholic beverages [g]	−0.449 (0.059)	−0.048 (0.122)	**−0.179 (0.002)**	−0.014 (0.145)	−0.004 (0.904)	−0.01 (0.185)	−0.003 (0.817)
Beverages with extrinsic sugar [g]	−0.125 (0.592)	−0.046 (0.129)	−0.084 (0.15)	−0.003 (0.731)	−0.002 (0.923)	−0.008 (0.307)	−0.009 (0.459)
Dairy products [g]	−0.211 (0.287)	−0.022 (0.404)	−0.005 (0.924)	−0.008 (0.345)	−0.028 (0.123)	−0.006 (0.362)	−0.009 (0.376)
Fermented dairy products [g]	−0.342 (0.201)	−0.046 (0.19)	0.035 (0.61)	−0.014 (0.216)	−0.031 (0.209)	−0.007 (0.413)	−0.021 (0.144)
Butter [g]	−14.385 (0.131)	−1.261 (0.312)	0.904 (0.711)	−0.709 (0.068)	−0.979 (0.274)	−0.181 (0.566)	−0.529 (0.301)
Bread [g]	−0.67 (0.447)	−0.129 (0.259)	−0.099 (0.659)	−0.029 (0.417)	0.024 (0.774)	−0.019 (0.512)	−0.022 (0.648)
Whole grain [g]	1.075 (0.493)	−0.026 (0.897)	−0.395 (0.319)	0.044 (0.497)	0.104 (0.475)	0.019 (0.711)	0.05 (0.551)
Cereals [g]	−4.494 (0.251)	−0.767 (0.13)	−0.9 (0.366)	−0.123 (0.448)	−0.422 (0.254)	−0.115 (0.372)	−0.401 (0.052)
Sweet spreads [g]	−4.887 (0.171)	−0.758 (0.101)	−0.267 (0.771)	−0.208 (0.156)	−0.504 (0.146)	−0.101 (0.39)	−0.16 (0.405)
Eggs [g]	−1.464 (0.666)	−0.077 (0.862)	−0.861 (0.315)	−0.076 (0.585)	−0.224 (0.487)	−0.193 (0.078)	0.042 (0.815)
Meat [g]	−0.804 (0.586)	−0.261 (0.171)	−0.34 (0.363)	−0.051 (0.404)	0.104 (0.531)	−0.036 (0.453)	−0.016 (0.839)
Poultry [g]	3.504 (0.436)	0.363 (0.535)	−0.725 (0.525)	0.286 (0.117)	−0.146 (0.734)	−0.038 (0.795)	0.303 (0.203)
Fast foods [g]	−0.585 (0.1)	**−0.133 (0.003)**	**−0.272 (0.002)**	−0.027 (0.065)	−0.133 (0.59)	−0.005 (0.649)	−0.035 (0.068)
Savory spreads [g]	−2.786 (0.382)	−0.364 (0.379)	0.128 (0.874)	−0.178 (0.172)	0.221 (0.468)	−0.078 (0.455)	−0.045 (0.791)
Fish [g]	−2.218 (0.198)	**−0.646 (0.003)**	**−1.033 (0.016)**	−0.103 (0.145)	−0.565 (0.136)	−0.011 (0.844)	**−0.193 (0.033)**
Fruit [g]	−0.16 (0.41)	**−0.052 (0.035)**	**−0.145 (0.002)**	**0.093 (0.03)**	0.026 (0.261)	−0.003 (0.695)	−0.012 (0.246)
Vegetables [g]	−0.52 (0.092)	**−0.079 (0.048)**	−0.057 (0.475)	−0.02 (0.116)	0.051 (0.088)	< 0.001 (0.999)	**−0.033 (0.041)**
Rice/Pasta/Potatoes [g]	−0.391 (0.051)	**−0.082 (0.001)**	**−0.158 (0.001)**	**−0.017 (0.038)**	0.062 (0.564)	−0.002 (0.755)	**−0.023 (0.033)**
Highly processed carbohydrates [g]	**−0.921 (0.016)**	**−0.15 (0.002)**	**−0.214 (0.028)**	**−0.04 (0.01)**	−0.005 (0.927)	−0.009 (0.475)	**−0.045 (0.029)**
Sweets [g]	−1.867 (0.078)	−0.224 (0.104)	−0.275 (0.311)	−0.061 (0.163)	−0.033 (0.768)	−0.036 (0.311)	−0.069 (0.223)
Food with extrinsic sugar [g]	**−45.063 (0.048)**	**−0.208 (0.049)**	−0.219 (0.297)	−0.053 (0.116)	−0.072 (0.401)	−0.032 (0.241)	−0.068 (0.122)
Snacks [g]	−4.006 (0.245)	−0.435 (0.333)	−0.996 (0.254)	−0.081 (0.57)	−0.261 (0.47)	−0.14 (0.215)	0.024 (0.896)
Legumes [g]	−2.327 (0.237)	**−0.563 (0.025)**	−0.71 (0.154)	−0.114 (0.155)	0.138 (0.471)	0.002 (0.971)	**−0.225 (0.029)**
Nuts [g]	−12.738 (0.084)	**−2.305 (0.014)**	**−5.129 (0.005)**	−0.481 (0.113)	−0.968 (0.429)	−0.297 (0.222)	−0.571 (0.149)

**Table 4:** Mean values and standard deviations (SD) of different nutrients for the total population and subgroups (moderate periodontitis, severe periodontitis). Differences between the subgroups were assessed (*p*-values). Significant results are in bold.

	Total (SD)	Moderate Periodontitis (SD)	Severe Periodontitis (SD)	*p*-Value (*t*-Test)
Energy [kcal]	1818.85 (±1573.75)	2283.92 (±244.05)	1655.45 (±185.11)	0.219
Water [g]	4342.28 (±2310.96)	5325.3 (±2955.68)	3996.9 (±1971.17)	0.074
Protein [g]	77.88 (±58.23)	90.96 (±87.26)	73.29 (±44.65)	0.352
Fat [g]	65.26 (±57.78)	74.01 (±79.63)	62.18 (±48.92)	0.531
Carbohydrates [g]	212.28 (±190.56)	288.09 (±299.3)	185.64 (±129.46)	0.096
Total sugar [g]	110.84 (±84.7)	144.79 (±94.38)	98.91 (±78.95)	0.093
Intrinsic sugar [g]	74.67 (±56.48)	**106.82 (±74.12)**	**63.38 (±44.8)**	**0.016**
Extrinsic sugar [g]	36.16 (±44.68)	37.97 (±39.49)	35.53 (±46.86)	0.867
Fiber [g]	20.97 (±15.86)	**28.75 (±20.64)**	**18.24 (±13.07)**	**0.038**
Starch [g]	99.74 (±122.19)	139.91 (±221.57)	85.62 (±56.3)	0.171
Water-soluble fiber [g]	6.52 (±5.11)	**9.02 (±7.33)**	**5.64 (±3.82)**	**0.039**
Water-insoluble fiber [g]	13.84 (±10.72)	**19.18 (±13.96)**	**11.97 (±8.81)**	**0.035**
Saturated fatty acids [g]	30.56 (±27.59)	32.22 (±29.84)	29.97 (±27.16)	0.803
Polyunsaturated fatty acids [g]	9.23 (±10.11)	12.58 (±18.1)	8.06 (±4.96)	0.168
Short-chain fatty acids [g]	1.59 (±1.83)	1.62 (±1.22)	1.59 (±1.83)	0.940
Medium-chain fatty acids [g]	1.51 (±1.65)	1.66 (±1.64)	1.45 (±1.68)	0.702
Long-chain fatty acids [g]	57.39 (±50.61)	65.63 (±71.93)	54.49 (±41.57)	0.501
Alcohol [g]	7.86 (±16.54)	10.08 (±26.72)	7.08 (±11.48)	0.580
Vitamin A [µg]	1461.67 (±1227.32)	1695.9 (±1618)	1379.38 (±1072.78)	0.429
Carotene [mg]	5.9 (±6.33)	7.36 (±7.98)	5.39 (±5.68)	0.339
Vitamin D [µg]	4.44 (±5.23)	5.63 (±8.37)	4.02 (±3.63)	0.347
Vitamin E (equivalent) [mg]	9.48 (±10.49)	13 (±16.84)	8.24 (±7.01)	0.162
Vitamin B_1_ [mg]	1.14 (±0.75)	1.42 (±1.16)	1.03 (±0.53)	0.110
Vitamin B_2_ [mg]	1.81 (±1.151)	2.08 (±1.41)	1.71 (±1.05)	0.330
Niacin [mg]	16.37 (±12.7)	21.82 (±22.52)	14.46 (±6.01)	0.072
Pantothenic acid [mg]	5.58 (±3.91)	7.37 (±1.56)	4.94 (±0.48)	0.053
Vitamin B_6_ [mg]	1.78 (±1.33)	**2.43 (±2.09)**	**1.55 (±0.86)**	**0.039**
Biotin [µg]	52.38 (±33.71)	**68.78 (±40.14)**	**46.62 (±29.64)**	**0.040**
Folic acid [µg]	259.15 (±194.14)	331.66 (±225.88)	233.67 (±178.12)	0.119
Vitamin B_12_ [µg]	5.08 (±4.08)	5.96 (±6.37)	4.77 (±2.96)	0.373
Vitamin C [mg]	164.54 (±139.33)	**241.83 (±153.34)**	**137.39 (±125.19)**	**0.018**
Sodium [mg]	2315.52 (±2539.35)	3008.73 (±4623.53)	2071.97 (±1190.25)	0.257
Potassium [mg]	3308.51 (±2452.57)	**4463.34 (±3928.98)**	**2902.76 (±1546.21)**	**0.047**
Calcium [mg]	1282.48 (±798.19)	1349.25 (±745.72)	1259.02 (±824.39)	0.730
Magnesium [mg]	418.31 (±235.62)	519.94 (±342.71)	382.61 (±177.13)	0.070
Phosphorus [mg]	1386.95 (±981.76)	1591.65 (±1301.67)	1315.03 ± (852.46)	0.388
Iron [mg]	10.87 (±8.21)	13.66 (±13.13)	9.9 (±5.53)	0.158
Zinc [mg]	10.72 (±7.51)	12.6 (±12.1)	10.06 (±5.12)	0.298
Iodide [µg]	144.65 (±91.57)	183.77 (±138.41)	130.91 (±65.42)	0.073
Cholesterol [mg]	271.37 (±221.12)	322.27 (±332.19)	253.49 (±168.83)	0.340
Tea [g]	0.276 (±0.538)	**0.544 (±0.902)**	**0.181 (±0.295)**	**0.035**
Coffee [g]	0.278 (±0.283)	0.256 (±0.255)	0.286 (±0.295)	0.743
Non-alcoholic beverages [g]	0.095 (±0.125)	0.11 (±0.174)	0.09 (±0.106)	0.617
Water as a beverage [g]	1.654 (±1.439)	1.696 (±1.554)	1.64 (±1.418)	0.905
Alcoholic beverages [g]	0.07 (±0.13)	0.035 (±0.053)	0.083 (±0.147)	0.260
Beverages with extrinsic sugar [g]	0.069 (±0.091)	0.085 (±0.119)	0.063 (±0.08)	0.467
Dairy products [g]	0.17 (±0.13)	0.161 (±0.125)	0.174 (±0.133)	0.760
Fermented dairy products [g]	0.045 (±0.053)	0.067 (±0.056)	0.037 (±0.051)	0.081
Butter [g]	0.003 (±0.003)	0.001 (±0.001)	0.003 (±0.003)	0.082
Bread [g]	0.045 (±0.03)	0.036 (±0.028)	0.048 (±0.031)	0.215
Whole grain [g]	0.023 (±0.026)	0.025 (±0.024)	0.023 (±0.027)	0.816
Cereals [g]	0.007 (±0.01)	**0.013 (±0.015)**	**0.005 (±0.006)**	**0.011**
Sweet spreads [g]	0.005 (±0.006)	0.004 (±0.004)	0.006 (±0.007)	0.367
Eggs [g]	0.011 (±0.012)	0.011 (±0.017)	0.011 (±0.009)	0.932
Meat [g]	0.035 (±0.029)	0.025 (±0.019)	0.038 (±0.032)	0.160
Poultry [g]	0.012 (±0.009)	0.011 (±0.009)	0.012 (±0.009)	0.890
Fast foods [g]	0.026 (±0.025)	0.027 (±0.036)	0.026 (±0.02)	0.898
Savory spreads [g]	0.014 (±0.016)	0.01 (±0.012)	0.015 (±0.017)	0.337
Fish [g]	0.012 (±0.011)	0.013 (±0.012)	0.011 (±0.011)	0.638
Fruit [g]	0.181 (±0.213)	**0.289 (±0.287)**	**0.143 (±0.169)**	**0.032**
Vegetables [g]	0.13 (±0.123)	0.128 (±0.1)	0.13 (±0.131)	0.965
Rice/Pasta/Potatoes [g]	0.075 (±0.043)	0.08 (±0.591)	0.074 (±0.037)	0.671
Highly processed carbohydrates [g]	0.083 (±0.033)	0.075 (±0.042)	0.086 (±0.029)	0.288
Sweets [g]	0.029 (±0.023)	0.031 (±0.032)	0.028 (±0.019)	0.701
Food with extrinsic sugar [g]	0.041 (±0.028)	0.048 (±0.04)	0.039 (±0.022)	0.312
Snacks [g]	0.005 (±0.005)	0.004 (±0.003)	0.005 (±0.006)	0.459
Legumes [g]	0.015 (±0.016)	0.016 (±0.014)	0.015 (±0.017)	0.087
Nuts [g]	0.003 (±0.003)	0.003 (±0.003)	0.002 (±0.003)	0.737

**Table 5:** Comparison of the nutrients consumed by the total population as well as the different groups in relation to dietary recommendations from German Nutrition Society and WHO. The fields are colored by the following scheme: Values are highlighted in green when the recommended daily intake of an anti-inflammatory food is achieved or the threshold for a pro-inflammatory food is not exceeded. Conversely, values are highlighted in red when the recommended intake for an anti-inflammatory food is not met or the daily threshold for a pro-inflammatory food is surpassed. If the boxes are white, no clear assignment can be made.

	Reference	Relative Differences between Reference and Total (Reference as Baseline)	Relative Differences between Reference and Moderate Periodontitis (Reference as Baseline)	Relative Differences between Reference and Severe Periodontitis (Reference as Baseline)
Energy [kcal]	2250 kcal	−19.16%	1.51%	−26.42%
Water [g]	30 g/kg/day	85.69%	127.72%	70.92%
Protein [g]	0.8 g/kg/day	52.71%	78.35%	43.71%
Fat [g]	30% of daily energy intake	7.64%	−2.79%	12.68%
Carbohydrates [g]	<50% of daily energy intake	−6.63%	0.91%	−10.29%
Extrinsic sugar [g]	5% of daily energy intake	59.06%	33.00%	71.70%
Fiber [g]	>30 g/day	−30.09%	−4.17%	−39.20%
Saturated fatty acids [g]	<10% of daily energy intake	51.20%	26.97%	62.93%
Polyunsaturated fatty acids [g]	<7–10% of daily energy intake	−34.73%	−29.18%	−37.40%
Vitamin A [µg]	775.00	88.60%	118.83%	77.98%
Carotene [mg]	4.65	26.93%	58.28%	15.91%
Vitamin D [µg]	20.00	−77.81%	−71.85%	−79.90%
Vitamin E (equivalent) [mg]	12.50	−24.15%	4.00%	−34.08%
Vitamin B_2_ [mg]	1.15	57.06%	80.87%	48.70%
Niacin [mg]	13.00	25.93%	67.85%	11.23%
Vitamin B_6_ [mg]	1.50	18.40%	62.00%	3.33%
Biotin [µg]	40.00	30.94%	71.95%	16.55%
Folic acid [µg]	300.00	−13.62%	10.55%	−22.11%
Vitamin B_12_ [µg]	4.00	26.98%	49.00%	19.25%
Vitamin C [mg]	102.50	60.53%	135.93%	34.04%
Sodium [mg]	1500.0	54.37%	100.58%	38.13%
Potassium [mg]	4000.00	−17.29%	11.58%	−27.43%
Calcium [mg]	1000.00	28.25%	34.93%	25.90%
Magnesium [mg]	325.00	28.71%	59.98%	17.73%
Phosphorus [mg]	700.00	98.14%	127.38%	87.86%
Iodide [µg]	180.00	−19.64%	2.09%	−27.27%
Dairy [g]	300.00	−5.98%	−14.74%	−2.90%
Fermented dairy [g]	150.00	−37.31%	−29.85%	−39.93%
Coffee [g]	700.00	−41.19%	−32.54%	−44.24%
Water as a beverage [g]	1500.00	51.88%	66.94%	46.59%
Bread [g]	300.00	−73.77%	−77.84%	−72.34%
Eggs [g]	21.43	−13.01%	−12.32%	−13.25%
Fish [g]	31.50	−30.48%	19.30%	−47.97%
Fruit [g]	250.00	18.24%	105.63%	−12.47%
Vegetables [g]	400.00	−48.91%	−45.35%	−50.17%
Rice/noodles/potatoes [g]	205.00	−22.47%	39.58%	−44.27%
Legumes [g]	125.00	−80.65%	−77.26%	−81.85%
Nuts [g]	25.00	−79.18%	−67.08%	−83.44%

## Data Availability

Data will be available from the corresponding author upon reasonable request.
